# ATG13 dynamics in nonselective autophagy and mitophagy: insights from live imaging studies and mathematical modeling

**DOI:** 10.1080/15548627.2020.1749401

**Published:** 2020-04-22

**Authors:** Piero Dalle Pezze, Eleftherios Karanasios, Varvara Kandia, Maria Manifava, Simon A. Walker, Nicolas Gambardella Le Novère, Nicholas T. Ktistakis

**Affiliations:** The Babraham Institute, Babraham Research Campus, Cambridge, UK

**Keywords:** ATG13, autophagy, LC3, mathematical modeling, mitophagy, ULK

## Abstract

During macroautophagy/autophagy, the ULK complex nucleates autophagic precursors, which give rise to autophagosomes. We analyzed, by live imaging and mathematical modeling, the translocation of ATG13 (part of the ULK complex) to the autophagic puncta in starvation-induced autophagy and ivermectin-induced mitophagy. In nonselective autophagy, the intensity and duration of ATG13 translocation approximated a normal distribution, whereas wortmannin reduced this effect and shifted to a log-normal distribution. During mitophagy, multiple translocations of ATG13 with increasing time between peaks were observed. We hypothesized that these multiple translocations arise because the engulfment of mitochondrial fragments required successive nucleation of phagophores on the same target, and a mathematical model based on this idea reproduced the oscillatory behavior. Significantly, model and experimental data were also in agreement that the number of ATG13 translocations is directly proportional to the diameter of the targeted mitochondrial fragments. Thus, our data provide novel insights into the early dynamics of selective and nonselective autophagy.

**Abbreviations:** ATG: autophagy related 13; CFP: cyan fluorescent protein; dsRED: *Discosoma* red fluorescent protein; GABARAP: GABA type A receptor-associated protein; GFP: green fluorescent protein; IVM: ivermectin; MAP1LC3/LC3: microtubule-associated protein 1 light chain 3; MTORC1: mechanistic target of rapamycin kinase complex 1; PIK3C3/VPS34: phosphatidylinositol 3-kinase catalytic subunit type 3; PtdIns3P: PtdIns-3-phosphate; ULK: unc-51 like autophagy activating kinase.

## Introduction

Autophagy can be nonselective, when the cargo is generic cytoplasmic material, providing the cell with a mechanism for nutrient supply in periods of starvation [1–3], or selective, when it leads to the degradation of specific intracellular structures: damaged mitochondria, endoplasmic reticulum fragments, bacterial pathogens, etc., to form the basis for a crucial cellular quality control system [4–7].

The starvation-induced pathway utilizes a series of membrane re-arrangements after the inactivation of the protein kinase complex MTORC1 [8]. This change leads to the activation of the autophagy-specific protein kinase ULK complex, formed by the protein kinase ULK1 (or its homolog ULK2), and RB1 CC1/FIP200, ATG13, and ATG101 [9,10]. Once active, the ULK complex translocates to the tubulovesicular regions of the ER, which are characterized by the presence of vesicles containing the autophagy protein ATG9. On these sites, the class III phosphatidylinositol 3-kinase (PtdIns3 K) complex I, containing PIK3 C3/VPS34, also translocate to produce phosphatidylinositol-3-phosphate (PtsIns3P) and generate omegasomes from where autophagosomes emerge [11,12]. In addition to this linear sequence, we have also proposed a PtdIns3P-dependent positive feedback loop reenforcing ULK binding to membranes in the early phases of omegasome-ULK expansion [13]. PtdIns3P binds to WIPI proteins, which, in turn, bind to the ATG16L1 protein, linking the formation of the autophagosomal membrane to the complexes, and covalently modifying LC3/GABARAP – the protein that binds to autophagosome cargo – with phosphatidylethanolamine [14,15].

In the case of selective autophagy, additional receptor proteins are necessary to label the cargo and connect it with the forming autophagosome [6,16–19]. Triggering these receptors leads to the recruitment of the LC3/GABARAP proteins, enabling the engulfment of the targeted membrane [20,21]. It should be noted that, however, even in the absence of all LC3/GABARAP proteins, autophagic membranes in the process of engulfing cargo can be observed [22].

Mitophagy in tissue culture cells can be induced by a variety of methods, including a number of pharmacological compounds, usually affecting the mitochondrial membrane potential or metabolite balance of the mitochondrial lumen [23]. In contrast to most of these drugs, which require several hours to take effect, the anthelmintic lactone ivermectin (IVM) was also found to induce mitochondrial fragmentation and mitophagy within 30 min of treatment, making it an ideal tool in our recent study of mitophagy dynamics [24]. In that work, we observed that the translocation of the ATG13 protein to the forming mitophagosomes occurred in several waves resembling oscillations [24], something that we had not seen for the translocation of the same protein to starvation-induced autophagosomes. Here, we used complementary *in vitro* and *in silico* approaches to investigate the dynamics of ATG13 in the initiation of nonselective autophagy and ivermectin-dependent mitophagy. To be able to compare these two processes directly, we used spinning disk confocal microscopy to analyze the dynamics of the response, and the same quantitation protocols to follow the formation of ATG13-containing autophagy structures. Our imaging data were used to derive mathematical models of the two processes.

Data-driven mathematical modeling has been a valuable method for describing biological signaling networks and investigating novel regulatory mechanisms [25–27], including autophagy dynamics [28]. Mathematical models have also been proposed for the upstream signaling of autophagosome formation [29,30].

## Results

### A mathematical model for ATG13 dynamics during nonselective autophagy

**Figure 1.**
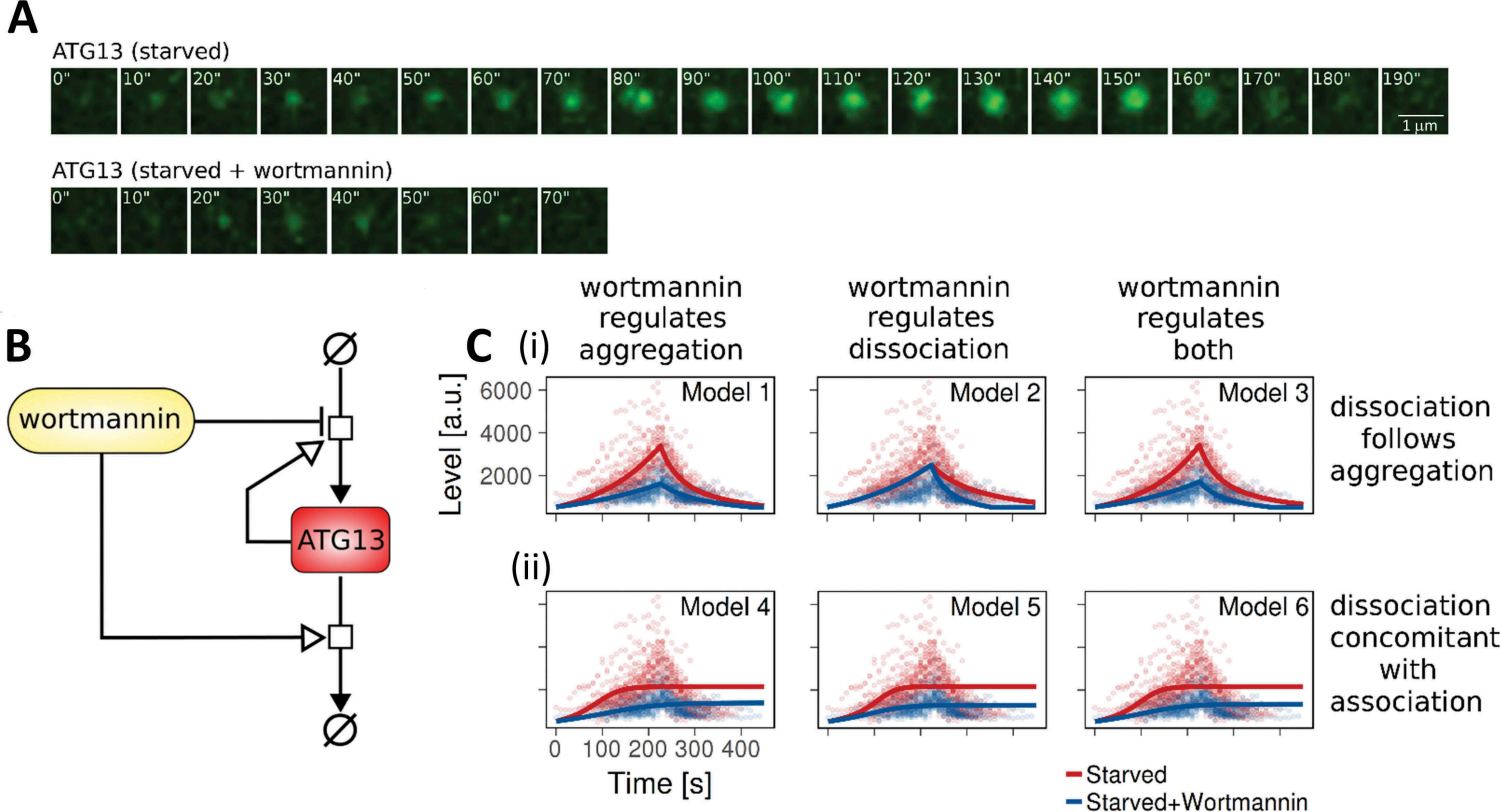
A mathematical model for nonselective autophagy. (A) Representative time-course images of one autophagy event involving the formation and disappearance of a GFP-ATG13 punctum. In the first row, cells are starved. In the second row, cells are starved and treated with wortmannin. (B) Schematic diagram of the mathematical model for nonselective autophagy. Please see the text for details. (C) Parameter fitting for the 6 model variants. (I) The switch between ATG13 accumulation and removal is regulated by events in Models 1–3. (ii) Models 4–6 are eventless. Wortmannin downregulates ATG13 accumulation (Models 1 and 4), ATG13 removal (Models 2 and 5), and both of the reactions (Models 3 and 6). Dots are experimental time points, whereas lines indicate model simulation after parameter estimation. Red indicates starvation; blue indicates starvation plus wortmannin

To generate a mathematical model of the earliest visible accumulation of autophagic structures in nonselective autophagy, cells stably expressing GFP-ATG13 were imaged during starvation, and data were collected in the presence and absence of the PI3 K inhibitor wortmannin (see also [13]). Representative time course images for one autophagy event are shown in Figure 1A. Wortmannin triggered a reduction of the ATG13 punctum intensity and lifetime, confirming the proposed PI3P-dependent positive regulation of ATG13 accumulation [13]. Autophagy events occurred in the cytoplasm asynchronously, and the weak signal at the beginning of the aggregation makes it difficult to determine an exact starting time point. Therefore, we synchronized these data using the ATG13 maximum intensity peak (see **Materials and Methods** for details).

We built a minimalistic model for the early steps of nonselective autophagy, where ATG13 aggregates and dissociates following mass-action reactions (Figure 1B). ATG13 accumulation and disappearance were non-linear, so we included a dependency for the process on the concentration of aggregated ATG13, with a partial reaction order *m*. To investigate the effect of wortmannin on these reactions, three models were investigated: 1) wortmannin regulating ATG13 aggregation, 2) wortmannin regulating ATG13 disappearance, and 3) wortmannin regulating both aggregation and disappearance. As ATG13 accumulation time courses were characterized by a rapid increase, followed by a steep decrease, we tested two additional hypotheses. In the first, the switch between ATG13 accumulation and removal was regulated by an event happening after *t* seconds from the initiation of the process. This delay was estimated from the imaging data. In the second, the reactions of ATG13 accumulation and removal happened simultaneously without any additional regulation. The complete description of the model is given in **Table S1-4** in BioRxiv (https://doi.10.1101/370114).

Parameter estimation was performed for the six model variants independently (see **Materials and Methods**), and the model fitting against the data is reported in Figure 1C. The identifiability of the model toward the core variables was extensively checked, finding clear optimal values for all parameters, as shown in **Table S3 and Fig. S1** in BioRxiv (https://doi.10.1101/370114). Physiological variations (a few percentages) of the values for most parameters did not affect the behavior of the model qualitatively. As the number of parameters is different according to the model, we compared their fit quality using the Akaike Information Criterion (AIC) [31], which penalizes models with a higher number of estimated parameters ([**Table S5** in BioRxiv {https://doi.10.1101/370114}]). The two models fitting the data featured wortmannin-regulated ATG13 accumulation and event-triggered disappearance. Since no significant differences were evident in the fitting of either models 1 and 3, we selected model 3, where wortmannin affects both aggregation and disappearance, without restricting the molecular mechanisms *a priori*, letting the parameter estimation to balance the extent of the two effects. This model was also the only one where all the parameters were identifiable with a 66% confidence level (**Table S3** in BioRxiv [https://doi.10.1101/370114]). Surprisingly, parameter estimation revealed a value of 1.01365 for the parameter *m* (confidence interval at 66%: [0.164715, 1.60609]) representing the partial reaction order of ATG13 complex accumulation on the already aggregated complex. This result suggested significant cooperativity for ATG13 (and likely ULK complex) accumulation.

### Wortmannin affected ATG13 peak time distribution in nonselective autophagy

**Figure 2.**
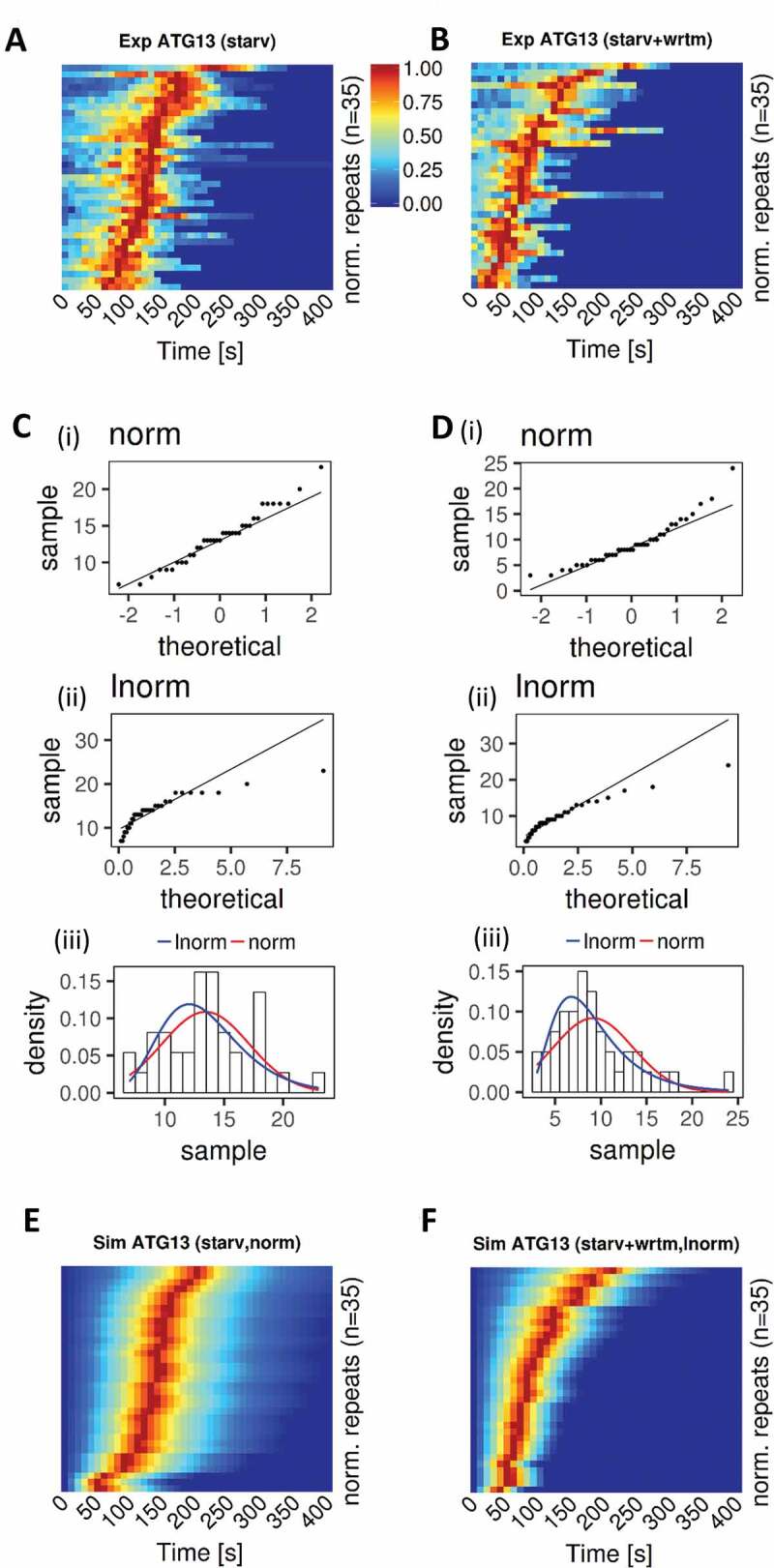
PtdIns3P inhibition upon wortmannin treatment shifts ATG13 peak time distribution from normal to log-normal. (A) Experimental kymographs of ATG13 upon starvation. For each group, n = 35 experimental repeats were selected, and the signal intensity of each repeat was normalized within [0,^1^] and indicated with the colors [blue, red]. Repeats were sorted by decreasing peak time. (B) Equivalent to panel A but for ATG13 upon starvation plus wortmannin. (C) Normality (I), log-normality (ii), and combination (iii) analysis of experimental peak times for ATG13 time courses upon starvation confirmed that the peak times were normally distributed. (D) Equivalent to panel C (i, normal; ii, lognormal; iii combination) but for ATG13 upon starvation plus wortmannin. Peak times for ATG13 were log-normally distributed. (E) Simulated kymographs of ATG13 upon starvation. ATG13 peak time (parameter *t*) was sampled from a normal distribution. A total of n = 35 simulated repeats were computed, and the signal intensity of each repeat was normalized within [0,1] and indicated with the colors [blue, red]. Repeats were sorted by decreasing peak time. (F) Equivalent to panel E, but for ATG13 upon starvation plus wortmannin. ATG13 peak time (parameter *t*) was sampled from a log-normal distribution using meanlog and sdlog

Since PtdIns3P feedback during initiation of autophagosome formation had been suggested [13], we analyzed the effect of wortmannin on ATG13 accumulation (Figure 2 A,B). First, we verified that there was no significant correlation between the signal peak times and initial signal intensities, which could have pointed to a possible misidentification of the start of aggregation and, therefore, of signal peak times (**Fig. S2**). The signal intensity of each experimental time course repeat was then normalized within [0,^1^]. Sorting repeats by decreasing peak times (dark red points in Figure 2 A,B) suggested that the addition of wortmannin changed the probability distribution. Q-Q plots for ATG13 peak times upon starvation (Figure 2C) and starvation plus wortmannin (Figure 2D) versus normal or log-normal distributions suggested that the peak times for ATG13 fluorescence signal upon starvation tended to approximate a normal distribution, whereas wortmannin shifted the peak times to approximate a log-normal distribution. We statistically tested whether the peak times were normally distributed. The Shapiro-Wilk test could not reject the null hypothesis that the starvation without wortmannin was normally distributed (*p*-value: 0.432; skewness: 0.312, excess kurtosis: −0.251) but did reject the null hypothesis for the starvation plus wortmannin (*p*-value: 0.004; skewness: 1.193, excess kurtosis: 1.716). If the data are distributed log-normally, the logarithms of the data are expected to be normal. The Shapiro-Wilk test using the logarithms of the starvation plus wortmannin group could not reject the null-hypothesis (*p*-value: 0.855), suggesting that this data set could be normally distributed. The skewness and excess kurtosis for this set were very close to 0 (skewness: −0.116, excess kurtosis: −0.198), indicating that the distribution is almost normal and supporting the original conclusion that the starvation plus wortmannin group is log-normally distributed. Using model 3 described above, we simulated populations of nonselective autophagy events with and without wortmannin, where the peak time (parameter *t*) was sampled from a normal or log-normal distribution, respectively (Figure 2E and Table S6 in BioRxiv [https://doi.10.1101/370114]). Those simulations adequately reproduced the experimental data and suggested that our model can be used to describe the ATG13-dependent initial step of autophagosome formation.

### A mathematical model for mitophagy

We decided to set up live imaging experiments of ATG13 and a mitochondrial marker after treatment with IVM to compare and contrast our data from nonselective autophagy to mitophagy [24]. We ensured that ATG13 colocalized with the targeted mitochondrion and formed, at least for part of the sequence, a quasi-ring structure around the mitochondrion at some point to consider only mitophagy events in our analysis. With these restrictive conditions, we were able to find 23 time courses (Fig. S3). Of highest concern to be excluded were events of nonselective autophagosome formation in close apposition to the mitochondria surface [32].

**Figure 3.**
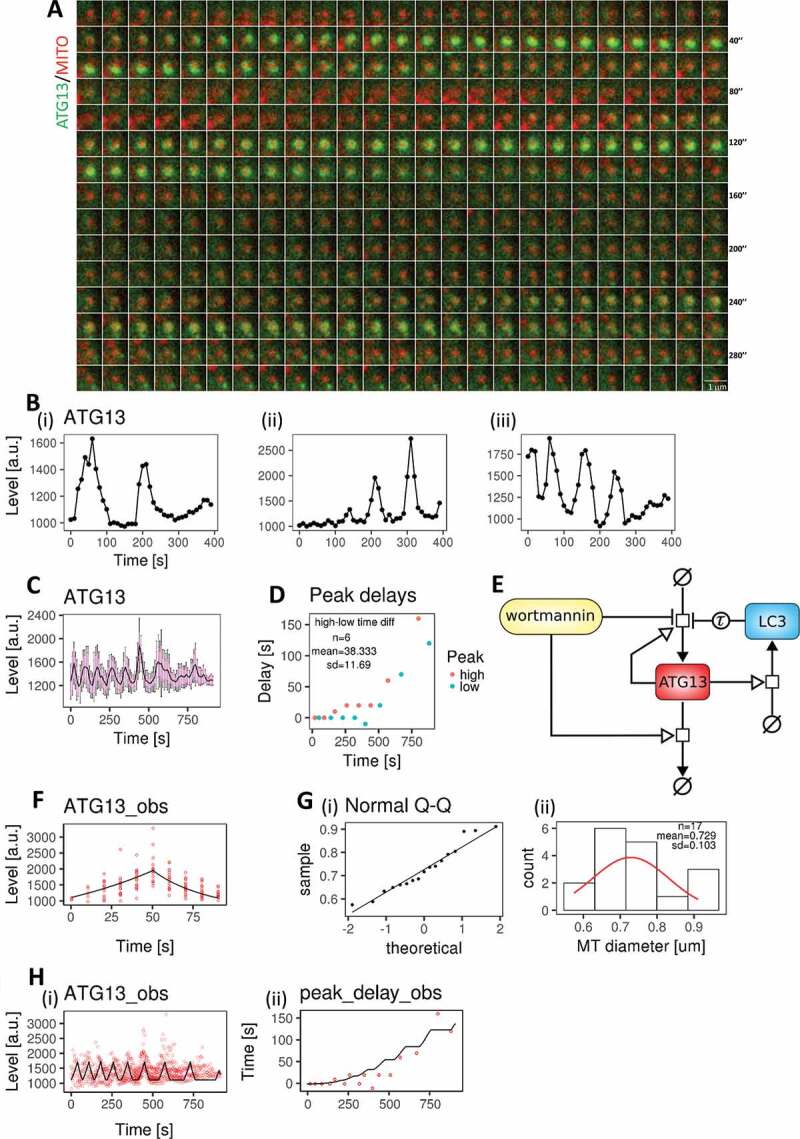
A mathematical model for mitophagy. (A) Live imaging of GFP-ATG13 (green) translocations on a targeted mitochondrion (red) showing sequential translocations. Each frame is 0.8” and the entire sequence is shown. This wide-field imaging was not used for the quantitations but provided a more continuous view of the translocations. (B) Three representatives of regularized quantified time courses up to 400 s (i–iii). These plots are derived from spinning disk confocal images. (C) ATG13 mean time course (black line) after data synchronization, filtering, and regularization. The mean is shown with the confidence interval of the mean at 95% confidence level (magenta bars) and 1 standard deviation (black bars). (D) The delays of the upper and lower aggregation peaks, indicating how these increased over time exponentially. Mean and standard deviation of the time differences between upper and lower peaks are reported as annotations. These statistics define the model parameter *t*. (E) Model diagram for mitophagy events. This model extends the diagram in Figure 1B by including LC3 and the events for regulating the delays between aggregations. The concentration of LC3 was calculated before the event starting ATG13 accumulation was triggered. Therefore, LC3 inhibits ATG13 accumulation after a delay (Tau). (F) Model fitting for ATG13 kinetic rate constants using the 1st aggregation data set. The experimental data and model simulation are represented with red circles and a black line, respectively. (G) MT diameter density (I) and QQ plot (ii) against a normal distribution. 17 mitochondrial diameters were measured and analyzed. The reported mitochondria diameter mean and standard deviation define the model parameter *MT_diam*. (H) Model fitting for ATG13 (i) and the delay between aggregations (ii) using the data sets in panel C. The parameters *t* and *MT_diam* were fixed to their mean value during this stage of parameter estimation

In contrast to the nonselective autophagy, where there was a single peak of ATG13, mitophagy ATG13 appeared to join and leave the targeted mitochondrion several times, revealing oscillatory dynamics (Figure 3A shows an example from a live imaging experiment at high frame rate, see [24]). Time courses were quantified, synchronized, filtered, and regularized (see **Fig. S4 and Materials and Methods** for details). Figure 3B shows 3 such examples and Figure 3C shows the final mean time course from the 17 datasets, including a confidence interval of the mean at 95% confidence level and 1 standard deviation. From the mean time course, we identified the peaks and troughs for each aggregation event, and we showed that the delay between events tends to increase exponentially along the time course (Figure 3D).

Like nonselective autophagy, the ULK complex initiates a cascading mechanism that leads to mitochondrion engulfment by the LC3-covered membrane. To explain the increasing delays between repeated peaks of ATG13, we hypothesized that ULK complexes could only aggregate in regions of the mitochondrion surface not already covered by LC3-containing membranes. In this view, as engulfment progresses, the portion of the mitochondrial surface not covered by LC3 decreases, and the probability of a new ATG13 translocation event decreased. This process would continue until the whole mitochondrion has been engulfed by LC3-containing membranes. We extended the model developed for nonselective autophagy to include LC3 (Figure 3E), whose accumulation is driven by the presence of ATG13-containing complexes (see also [13]), and it, in turn, feeds back on those complexes by decreasing the probability of initiating a new aggregation event. This conclusion is consistent with the extensive live imaging data we recently reported [24]. The complete description of the model is given in **Table S7-10** in BioRxiv (https://doi.10.1101/370114).

This model was fitted with the mitophagy time course data. First, to parameterize ATG13 aggregation and disappearance, we extracted the first peak of each time course and synchronized them, as we did for the nonselective autophagy data set (Figure 3F). The identifiability of the model toward the core variables was extensively checked, finding clear optimal values for most parameters, as shown in **Table S9 and Fig. S3** in BioRxiv (https://doi.10.1101/370114). Exceptions were the parameters related to the production rate of LC3 and the duration of engulfment that are obviously linked. Physiological variations (a few percentages) of the values for the most parameters did not affect the behavior of the model qualitatively, but it slightly changed the dynamics of the oscillations. See **Materials and Methods** for details. Parameter estimation revealed that the ATG13 kinetic rate constants were not significantly different between the mitophagy model (parameters: kprodATG13 = 0.0114, CI95 = [0.0099, 0.0127]; kremATG13 = 0.0114, CI95 = [0.0010, 0.0139]) and the nonselective autophagy model (parameters: kprodATG13 = 0.0082, CI95 = [0.0052, 0.0193]; kremATG13 = 0.0029, CI95 = [0.0007, 0.0183]). We then estimated the remaining model parameters using the complete ATG13 time course data set. In the model, there are two parameters sampled from random variables. The first is the time between the start of an aggregation event and the peak of ATG13 intensity, which is sampled at each aggregation (parameter *t* as in the nonselective autophagy model). In line with nonselective autophagy (Figure 2C), we assumed a normal distribution for ATG13 peak times. We directly extracted the information needed to compute *t* from the time differences between the upper and lower peaks in Figure 3D. Detailed statistics are reported in **Table S11** in BioRxiv (https://doi.10.1101/370114). The second parameter is the mitochondrial diameter, which is randomly sampled at the beginning of each time course from the distribution of measured mitochondrial diameters. By analyzing the mitochondrial diameters from the imaging time courses, we found that the population approximated a normal distribution (Figure 3G and S2). In agreement with this observation, the Shapiro-Wilk statistical test could not reject the null hypothesis that these data were normally distributed (*p*-value: 0.379; skewness: 0.378, excess kurtosis: −1.061). In order to fit the mean experimental time course (Figure 3C), we fixed the values of time to peak and mitochondrial diameter to the mean of their experimental distributions, as reported in Figure 3 D,G (second plot). Figure 3H shows the fitting between the model and the data. Details for the parameter estimation and identifiability are reported in the **Materials and Methods**. Parameter estimation gave an insight into the increasing delay between aggregations. In particular, the parameter *p* determining the exponential increase for the delay was estimated to be ~2.79. This cubic growth is consistent with the idea that each aggregation event represents the coating of the target by both ATG13 and LC3 and that as more of the area will be covered, there will be a delay until the next available region is found and the LC3-modifying machinery translocates there. We tested for this in the next section.

### Mitochondrial fragment diameter determines the number of ATG13 translocations

**Figure 4.**
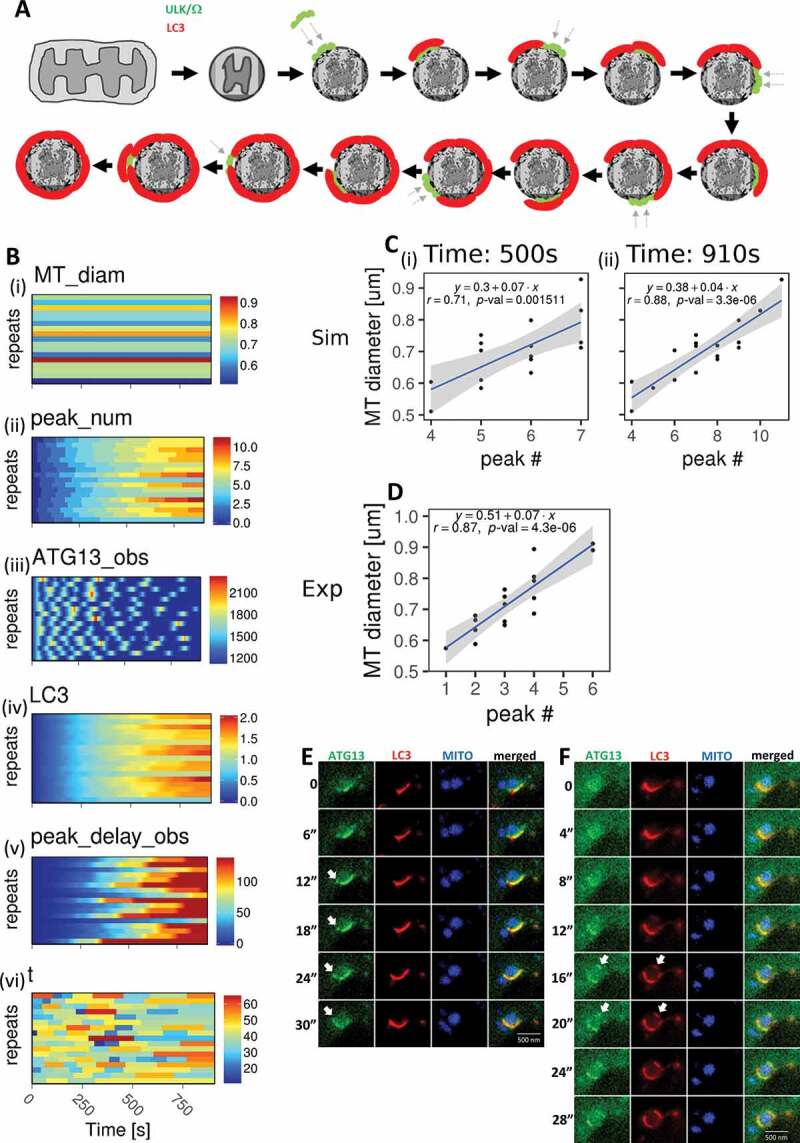
ATG13 sequential translocations depend on the mitochondrial size. (A) Proposed model for the sequential translocations of ATG13 on the targeted mitochondrial fragments. After fragmentation and damage, the early autophagic machinery represented by the ULK complex and omegasomes assembles on a piece of the mitochondrion surface (green elements) to be followed by LC3 membrane formation (red). This process is repeated 6 times until the LC3 membrane fully engulfs the mitochondrial fragment. Arrows indicate novel translocations to a new mitochondrial area. (B) Each plot represents 17 stochastic time courses, where time is the x-axis, the y-axis represents the 17 repeats (from top to bottom, each line a separate event), and the colored scale represents the intensity, with an arbitrary unit. (I), mt_diam: mitochondrial diameter, which does not change along the time course, so for each repeat, the mitochondrial diameter is flat, see also Fig. 3 G. (ii), peak_num: number of ATG13 aggregation peaks, which increase along the time course. (iii), ATG13_obs: the observable variable for ATG13. (iv) LC3: this is a model prediction for the LC3 time course for each of the 17 repeats (depicted horizontally). (V), peak_delay_obs: another observable variable, which is the delay between aggregation peaks and is modeled stochastically based on the experimental data. (vi), t: this is the time taken by ATG13 to reach an aggregation peak from the beginning of an oscillation, see also Fig 3D. (C) The model predicted a linear positive correlation between the mitochondrial diameters and the number of ATG13 aggregations at 510 s (i) at the end of the simulation at 910 s (ii). The fitting line is indicated in blue, whereas its confidence interval in gray. (D) Experimental relationship of mitochondrial diameter and number of ATG13 aggregations, reporting very similar correlation to the simulation in C. (E, F) Frames from live imaging of cells expressing GFP-ATG13, CFP-LC3, and drRED-MITO and induced for mitophagy by IVM treatment. Two separate time points are shown (panels E and F), where new domains of ATG13 and LC3 form away from the main region engulfing the targeted mitochondrial fragment (white arrows). Note that for clarity, the colors of LC3 and MITO are switched in these images. Bar: 500 μm

According to our hypothesis, each occurrence of ATG13 translocation (aggregation) promoted a partial coverage of a mitochondrion by LC3-containing membranes, and this process terminated when the whole mitochondrion became engulfed (see Figure 4A). According to this, the increasing delay between observed ATG13 peaks was due to two phenomena: 1) the time spent by ATG13 (as part of the ULK complex) searching a region on the mitochondrion surface, which had not yet been covered by LC3 membrane, and 2) the translocation of the LC3-modifying machinery. Under the assumption that the ULK complex drives engulfment by LC3, the latter delay should be constant over time as no random search is involved. Therefore, our model explicitly represents only the ATG13-dependent delay.

Using the parameters estimated above, we simulated 17 mitophagy events with the parameter *MT_diam* – determining the mitochondria diameter within the model – sampled from a normal distribution inferred from the data at the beginning of each simulation (see distribution parameters in Figure 3G plot 2 **and Table S10** in BioRxiv [https://doi.10.1101/370114]). The parameter *t*, setting the time between the beginning and the maximum signal of each ATG13 aggregation event, was also sampled from a normal distribution inferred from the data at the beginning of each ATG13 accumulation (see distribution parameters in Figure 3D **and Table S10-11** BioRxiv [https://doi.10.1101/370114]). The single simulation repeats for the main model readouts are shown in Figure 4B. The oscillations for a population of mitophagy events tended to lose synchronization over time due to the stochastic increase in delay between peaks. The normal distribution of aggregation duration means that the quantity of the LC3-membrane for each aggregation event driven by the ULK complex will also be normal. This result suggested that the size of a mitochondrion could determine the total number of accumulations needed to fully engulf it. Therefore, we analyzed whether there was a correlation between the number of ATG13 peaks and the sampled mitochondrial diameters. This analysis was conducted at the end of the simulation (910 s) and in the middle (500 s). Remarkably, the simulated data predicted a significant linear positive correlation (r = 0.88, *p*-value = 3.3e-0.6) between the number of ATG13 aggregations and the diameters of the corresponding engulfed mitochondria at the end of the simulation (Figure 4C). To test this prediction, we counted the number of peaks for the 17 repeats in our experimental data and plotted them against the corresponding mitochondrial diameters. A significant linear positive correlation was again obtained (r = 0.87, *p*-value = 4.3e-06) (Figure 4D).

Our model (Figure 4A) also predicted that translocation of ATG13 to the targeted mitochondrial fragment would be discontinuous and would be temporally and spatially related to the translocation of the LC3 protein. To examine this experimentally, we imaged by spinning disk confocal microscopy and using near-continuous image acquisition, cells expressing GFP-ATG13, CFP-LC3, and dsRED-MITO during IVM treatment. The spatial resolution of most mitophagy events was not sufficient to observe the separation of ATG13 and LC3 with clarity. However, we were able to examine some events involving large mitochondrial fragments, and, in those cases, it was easy to see the ATG13- and LC3-containing membranes forming discontinuously. Figure 4 E,F show two examples from the same targeted mitochondrion at different time points, where new domains of ATG13 and LC3 formed away from the main engulfed structure (white arrows).

## Discussion

In recent years, we and others have described the early membrane re-arrangements involved in the initiation of autophagy in some detail [33–35]. It is very clear from that work that the process is extremely complicated. It involves a series of hierarchical translocations of the relevant components on and off the forming autophagosomes, and it uses a large number of proteins and protein complexes. Here, we have focused on one of the earliest steps in this pathway, the translocation of the ATG13 protein to the forming autophagosomal structure, and used mathematical models to gain insight into the mechanisms that regulate it. Our work comes with the caveat that it relies on moderate overexpression of the relevant reporters, and, to the extent that it can be done, we have tried to relate our data to the endogenous proteins. For example, we have shown that the GFP-ATG13 protein used here co-localizes perfectly with endogenous ULK complex proteins RB1CC1, ULK1, and ATG101 [11,13] in various autophagy-inducing conditions, whereas immunoprecipitated GFP-ATG13 contains all subunits of the ULK complex (unpublished). We are, therefore, confident that the possibility of overexpression artifacts is minimal.

The formation of the ATG13 puncta in nonselective autophagy is characterized by a single event of accumulation. In our model, the cooperative aggregation of ATG13 is represented by the model parameter *m*, thus increasing the partial order of the ATG13 species for the reactions of accumulation and disappearance. Model parameter estimation partially identified this parameter and calculated a positive value, supporting the hypothesis of a positive feedback loop, thus increasing and decreasing the rate of ATG13 accumulation and removal, respectively. Such cooperativity could be explained, for instance, by a surface effect: the larger the ATG13 (and by extension the ULK complex) aggregates, the more sites there are for aggregation or disassembly. The second order (m = 1 plus the order of [ATG13]) could be consistent with a 2D (disk) rather than a 3D (sphere) expansion of the initiation apparatus.

Wortmannin-dependent inhibition of the PIK3 C3/VPS34-containing complex, which regulates PtdIns3P production, led to a decrease in the duration and intensity of ATG13 aggregations and a change in the probability distribution of ATG13 peak times from normal to log-normal. One possible explanation is that the inhibition of PtdIns3P synthesis removes external inputs on ULK complex accumulation, letting the process be driven only by complex stability. Without input from external factors, many natural phenomena relating to frequencies, for example, in this case, the kinetics of ATG13 disappearance (Figure 2D), can be described and explained by a log-normal distribution [36,37].

In contrast to nonselective autophagy, during mitophagy ATG13 accumulation and disappearance were more complex and displayed oscillatory dynamics (Figure 3A). According to our hypothesis, each event is characterized by ATG13 aggregation on an LC3-free subsurface on the targeted mitochondrion and drives the subsequent coverage by the LC3 membrane of this region (Figure 4A). Using time-course data analysis and mathematical modeling, we showed that these events delayed exponentially along the time course. In addition, model simulations predicted, and experimental data confirmed that the number of sequential translocations (oscillations) is proportional to the mitochondrial diameter. These findings indicated that mitophagy is dependent on the target structure and that the ULK complex, as exemplified by the ATG13 protein, is a key component for this process. How does this complex find an LC3-free subsurface on the mitochondrion? A potential explanation could be that the ULK complex can only accumulate on a region on the mitochondrion where mitophagy receptors can be detected. In this view, a region partially engulfed by LC3 would prevent the binding between the ULK complex and the mitophagy receptors.

Our study also analyzed the delay between ATG13 sequential translocations. Model parameter estimation revealed that this delay increases almost cubically with time. Given that the complex covers a surface, the delay between each step of partial engulfment would be expected to grow quadratically if no delays were involved. However, as the number of ATG13 translocations inherently depends on the time it takes the LC3 machinery to partially engulf the targeted mitochondrion, it appears clear that the translocation and engulfment of the LC3 machinery onto the mitochondrion surface adds a delay, and that our model calibration estimates the sum of the ATG13- and LC3-dependent delays. We predict that inhibiting LC3 lipidation would reduce the increase of delay, causing more frequent and more regular oscillations, as well as a slower engulfment by LC3. Our future work will attempt to introduce additional components to our imaging experiments to expand the scope (and complexity) of these models.

## Materials and methods

### Cell cultures

HEK-293 cells (ATCC CRL-1573) stably expressing GFP-ATG13 have been described before [13]. Immunofluorescence microscopy was performed, as described [38]. Mitophagy experiments were performed, as described [11,24].

### Raw data acquisition

Live cell imaging was performed, as described [11,24]. Cells were imaged in a closed dark chamber suffused with 5% CO_2_ and at 37°C. IVM (Sigma-Aldrich, I8898) or PP242 (Sigma-Aldrich, P0037) were added for 30 min before the start of image acquisition, and wortmannin (Sigma-Aldrich, W1628) was added simultaneously with the other compounds. The movies from which we derived the data were acquired over a 3-year period by 3 different operators, and no data set was used exclusively from a single imaging session done in a single day.

Images were acquired using a spinning disk confocal microscope, comprising Nikon Ti-E stand (MEA53100), Nikon 100 × 1.49 NA objective (MRD01990), Yokogawa CSU-X scan head (CR-CSUX-M1 N), Andor TuCam splitter (TR-DCIS-CMT) and Andor iXon 897 EM-CCD cameras (DU-897E-CS0-#BV-500). Using the splitter and 2x cameras enabled GFP (ATG13) and mCherry (mitochondria) images to be captured simultaneously. Images comprising 512 × 512 160 nm pixels were taken using a 200–300 ms exposure time, with z-stacks acquired at 10 s intervals. Each stack covered an approximately 6 μm range, with the step interval set at 500–700 nm to ensure optimal sampling. Maximum intensity projections of cropped regions were created using FIJI [39]. Imaris software (Bitplane, Oxford Instruments) was used to identify ATG13-positive puncta and measure spot intensity over time. Briefly, the “Spots” segmentation tool was used to analyze cropped image sequences, setting the estimated object diameter to 1 μm and using automated thresholding of the “Quality” parameter for object filtering. Tracking was done using the autoregressive motion algorithm setting a maximum distance of 3 μm and a maximum gap interval of 1 frame. Tracks were then filtered using time, typically leaving one object to measure for the entire duration of the cropped image sequence. To generate the nonselective autophagy data set, raw confocal images were acquired from 8 cells. From these images, 37 nonselective autophagy events were selected upon starvation and 40 events upon starvation plus wortmannin, measuring ATG13 as readout. As autophagy events were triggered within the cytoplasm at different time points, we synchronized the time courses based on their maximum intensity peak. To generate the mitophagy data set, raw confocal images were acquired from 25 cells, and 23 events were selected, assuring that ATG13 (green channel) co-localized with a mitochondrion (red channel) and ATG13 formed a quasi-circle around the mitochondrion at some point within the time course. This strategy allowed us to exclude potential autophagosome formation on the mitochondrion surface. This selection of mitophagy events was then quantified (**Fig. S3**). Then, the time courses were manually synchronized, overlapping the delays between aggregations. To facilitate the synchronization, the time courses were splined, reducing the noise within the data (**Fig. S6** in BioRxiv (https://doi.10.1101/370114]). Once synchronized, the splined time courses were replaced with the original time courses (**Fig. S4A and S4B**). Six time courses were removed due to their highly irregular nature. The initial and late time points were also cut off in order to have at least three repeats for each time point (**Fig. S4C**). Finally, the time courses were regularized to eliminate the gradual signal decline typically occurring over time in fluorescence images (**Fig. S4D**). The final single regularized time courses are reported in **Fig. S8** in BioRxiv (https://doi.10.1101/370114).

Mitochondria diameters were extracted using ImageJ 1.51 [40]. At least 8 mitochondria diameter measurements were taken for each of the 17 time courses (**Fig. S2**). The averages of these measurements were then computed and summarized in Figure 3G.

### ATG13 nonselective autophagy model

Parameter estimation was performed using Copasi 4.22 [41] and SBpipe 4.20.0 [42]. Particle Swarm optimization algorithm, as implemented in Copasi, was configured as follows: iteration number = 1000, and swarm size = 100. SBpipe was configured to run 1000 parameter estimates using Copasi within the SGE cluster at the Babraham Institute (UK).

The mathematical description for the nonselective autophagy model, including its events and parameters, is reported in **Table S1-4** in BioRxiv (https://doi.10.1101/370114). Synchronized time courses for ATG13 upon starvation and starvation plus wortmannin were simultaneously used as data sets for parameter estimation. The parameters (*kprodATG13, kremATG13, m, kwrtm, t*) were estimated for the models 1–3. The same parameters except *t* were estimated for models 4–6, as these models do not include events. For each of the six model variants, a total of 1000 parameter estimates were independently calculated. The 75% of the best fits (reporting the lowest objective value) were selected for analysis. Profile Likelihood Estimation (PLE) analysis was executed at the confidence levels of 66%, 95%, and 99% (**Table S3** in BioRxiv [https://doi.10.1101/370114]). Due to the different number of estimated parameters, the AIC was used for ranking the model variants (**Table S5** in BioRxiv [https://doi.10.1101/370114]).

### ATG13 mitophagy model

The mitophagy model extended the nonselective autophagy model with the inclusion of LC3 and oscillatory dynamics with delay for ATG13. The mathematical description for the mitophagy model, including its events and parameters, is reported in **Table S7-10** in BioRxiv (https://doi.10.1101/370114). Parameter estimation for the mitophagy model was performed in two steps. First, the first aggregation of ATG13 time courses was extracted and synchronized on the peak of maximum signal intensity as for the nonselective autophagy data. This reduced data set was used for estimating the kinetic rate constants for ATG13 (*kprod_ATG13* and *krem*_*ATG13*), and, therefore, compute the slopes of ATG13 signaling independently of the complex oscillatory behavior (**Table S9 and Fig. S3A** in Bioarxiv [https://doi.10.1101/370114]). Second, the parameters regulating the repeated aggregations (*kprodLC3, kpeak, p*) were estimated in two rounds of parameter estimation and identifiability. The first round revealed that *p* and *kprodLC3* were identifiable, although *kprodLC3* minima lay on a defined plateau. Fixing *kprodLC3*, the interdependent parameter *kpeak* was estimated and identified in the second round of parameter estimation (**Table S9, Fig. S3B, and S3 C** in BioRxiv [https://doi.10.1101/370114]). The parameters *t* and *MT*_*diam*, representing the time before reaching an aggregation peak and the mitochondria diameter, were fixed to the mean of their normal distribution during the second stage of parameter estimation, respectively.

### Statistical analysis

The statistical and programming language R version 3.4.0 (The R Project for Statistical Computing, https://www.r-project.org/) was used to generate statistics and plots. AIC [31], as computed in SBpipe was used for selecting the nonselective autophagy model, which fitted the nonselective autophagy dataset. Shapiro-Wilk test was used for testing normality distribution. A batch correction using R package SVA [43] was applied to the intensities of LC3 in Figure 1A to make the repeats of the two experiments of comparable intensity levels.

## Supplementary Material

Supplemental Material

## Note on manuscript organization

The complete manuscript, including all tables and supplementary material used for the derivation of the mathematical model, has been deposited in BioRxiv (https://doi.10.1101/370114). Throughout the present manuscript, we cite the Bioarxiv supplementary submission explicitly, including the Table or Figure number, for all additional supplementary material not able to be included here due to space limitations.
