# Supplementary material for: ATG13 dynamics in nonselective autophagy and mitophagy: insights from live imaging studies and mathematical modeling
Source: Autophagy. 2020 Apr 22;17(5):1131–41. doi: 10.1080/15548627.2020.1749401 (PMC8143212; doi:10.1080/15548627.2020.1749401)
Supplement: Supplemental Material [file KAUP_A_1749401_SM1095.pdf]

Supplementary Information
for

ATG13 dynamics in non-selective autophagy and mitophagy: insights from live imaging studies and mathematical modelling.

Piero Dalle Pezze, Eleftherios Karanasios, Varvara Kandia, Maria Manifava, Simon A. Walker, Nicolas Gambardella Le Novère and Nicholas T. Ktistakis.

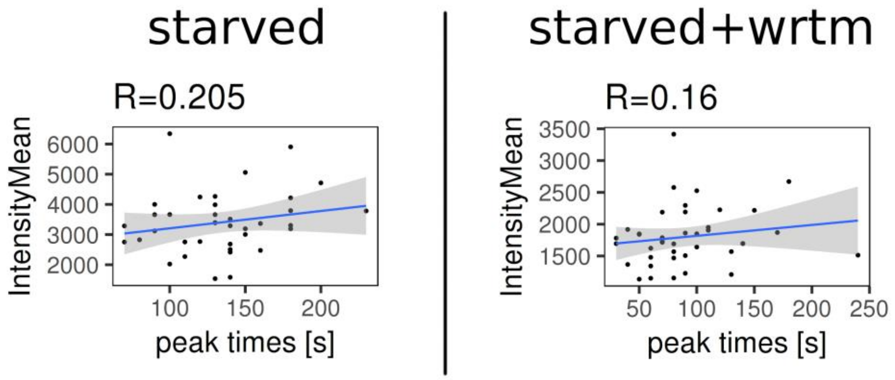

Supplementary Figure S1. Peak times did not correlate with signal intensities for the non-selective autophagy data.

No significant correlation was found between peak times and corresponding signal intensities for the starvation and starvation+wortmannin data sets in the non-selective autophagy.

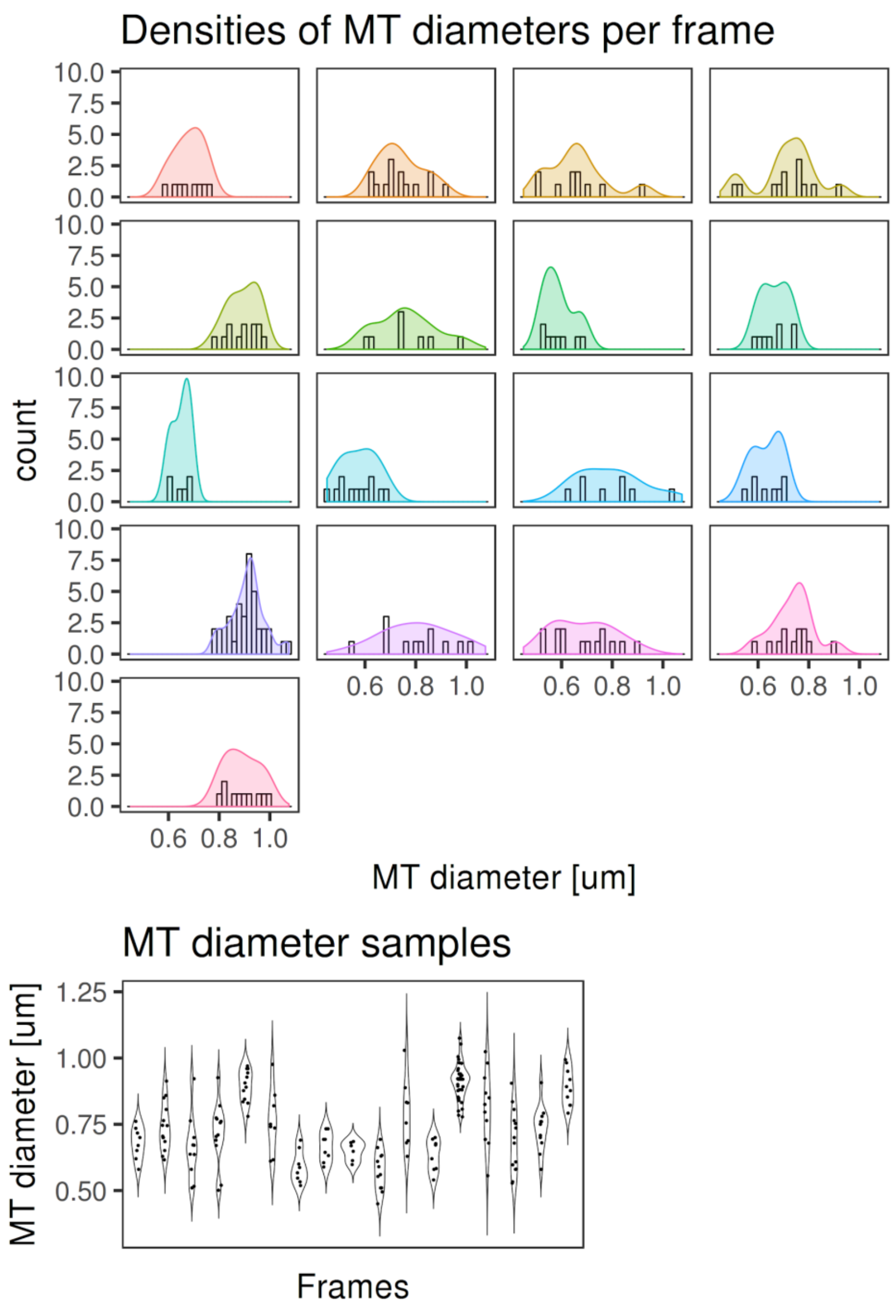

Supplementary Figure S2. Mitochondrial diameter measurements for each frame.

The diameter of the mitochondria engulfed by ATG13 was measured for each of the 17 time courses. The mean diameter for each frame was then computed.

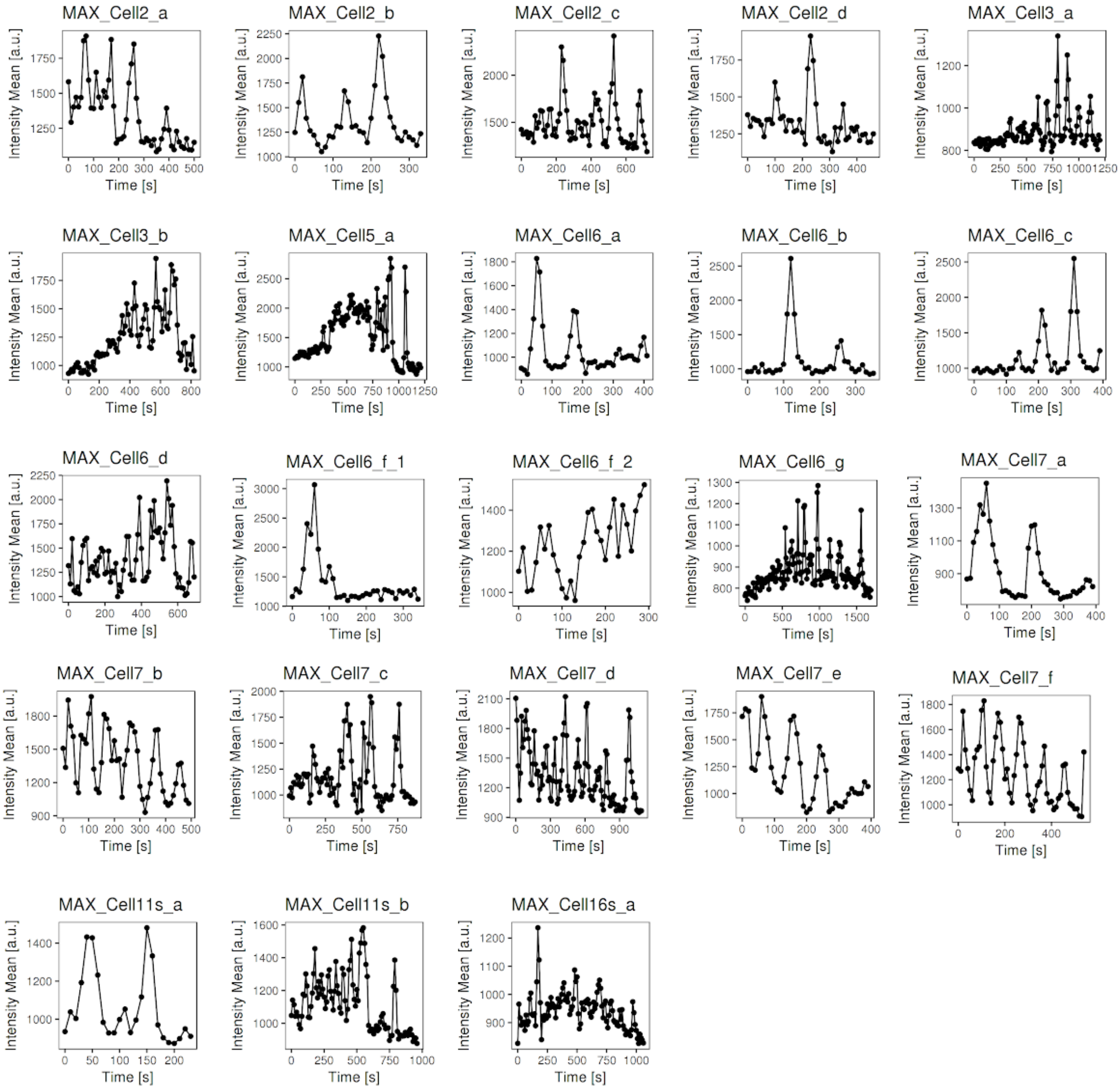

Supplementary Figure S3. Raw quantified time courses for ATG13 for the mitophagy model.

A total of 23 time courses were quantified from fluorescence large images. ATG13 accumulations/removals have similar lengths but the time between two peaks tend to increase over time. Due to this regularity, the time courses could have been synchronised.

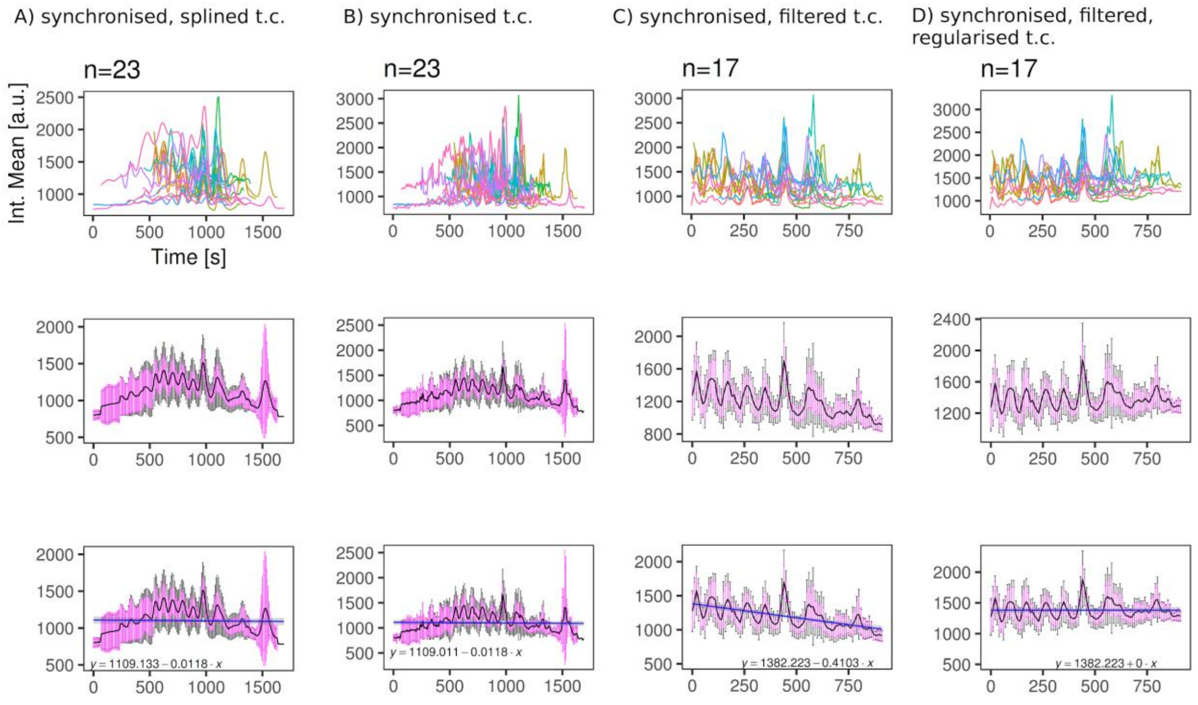

Supplementary Figure S4. Synchronisation and filtering of ATG13 time courses for the mitophagy model.

A) ATG13 splined time courses were synchronised by overlapping the delays between peaks.
B) The splined time courses were then replaced with the original ATG13 time courses.
C) Six time courses were removed because they contained too much noise in their signal. Initial and later signals were cut off in order to have at least 3 repeats for each time point.
D) The mean time course was regularised so that there was no signal decline over time. Each time course was adjusted based on this regularisation.
